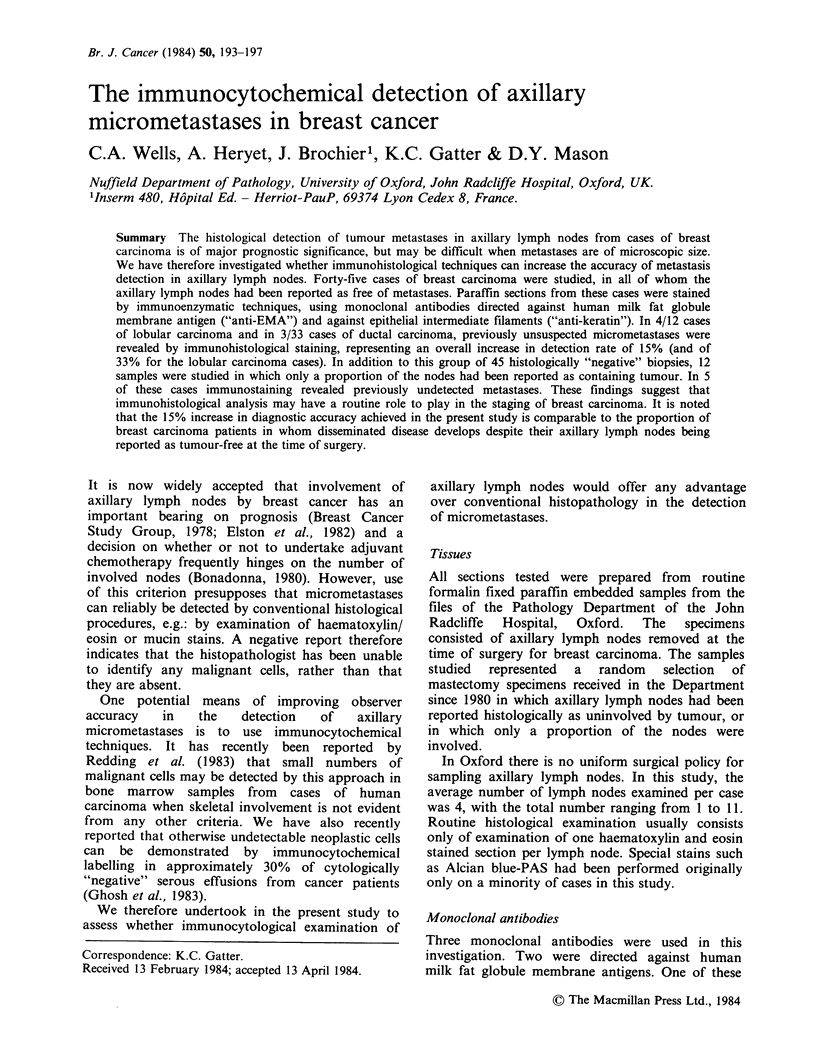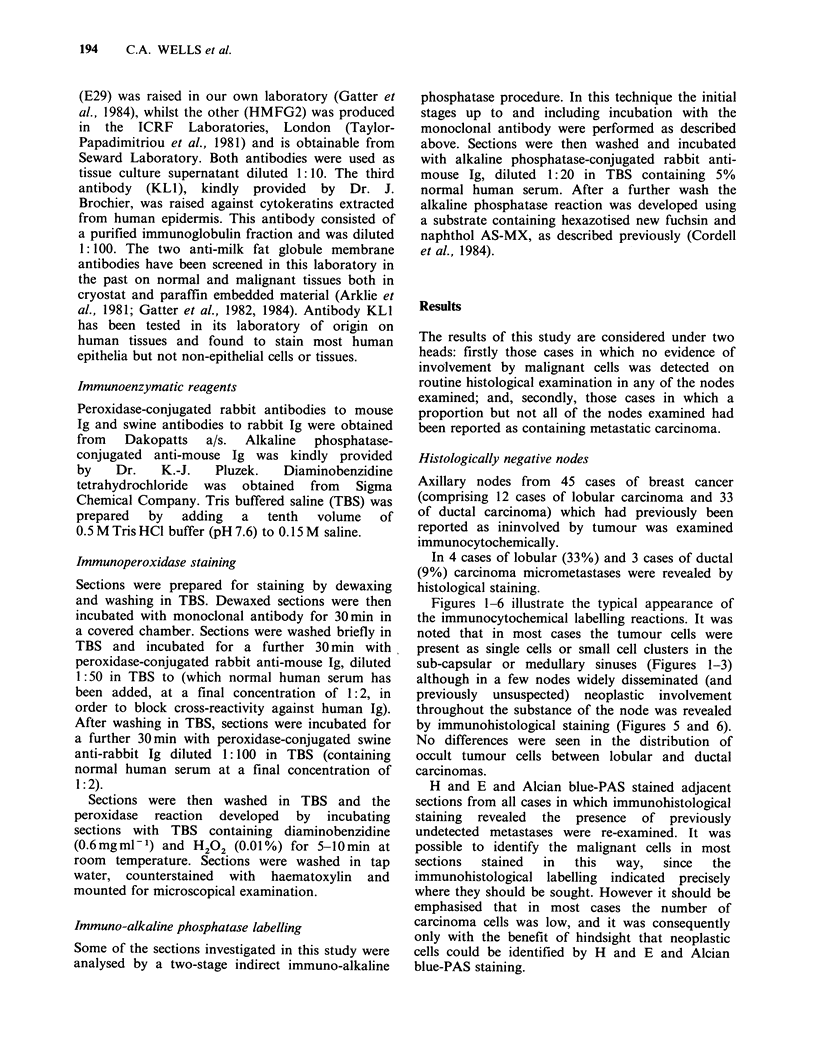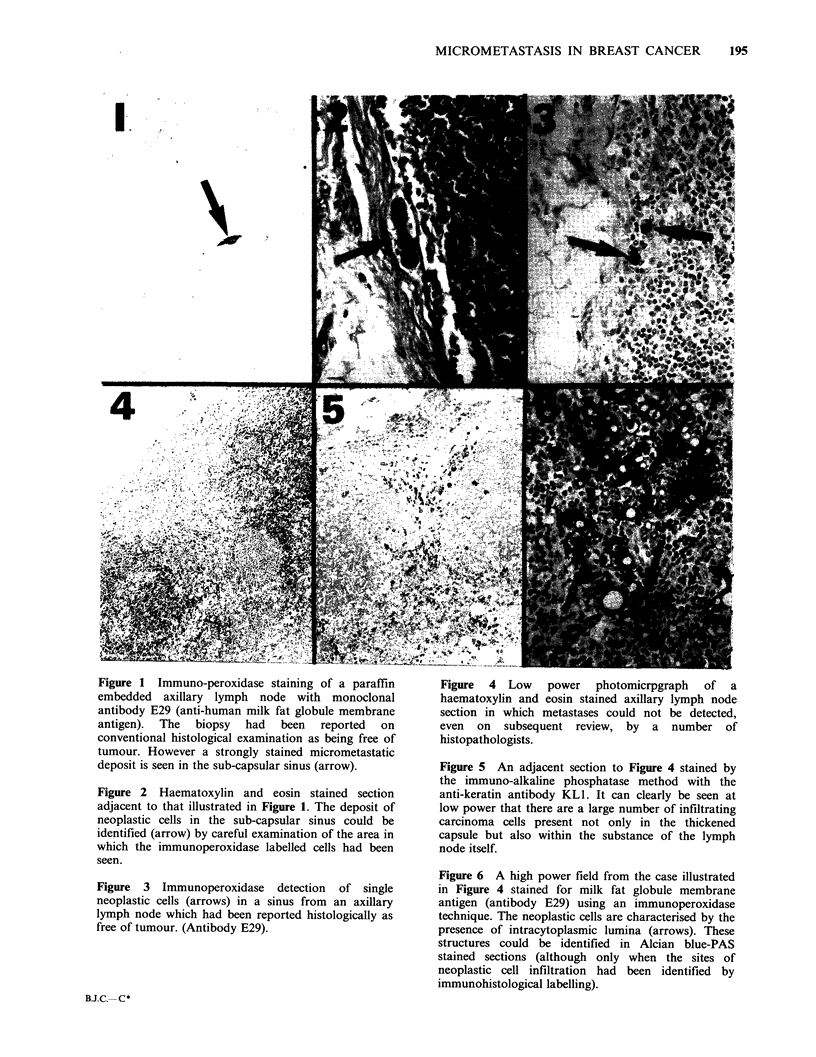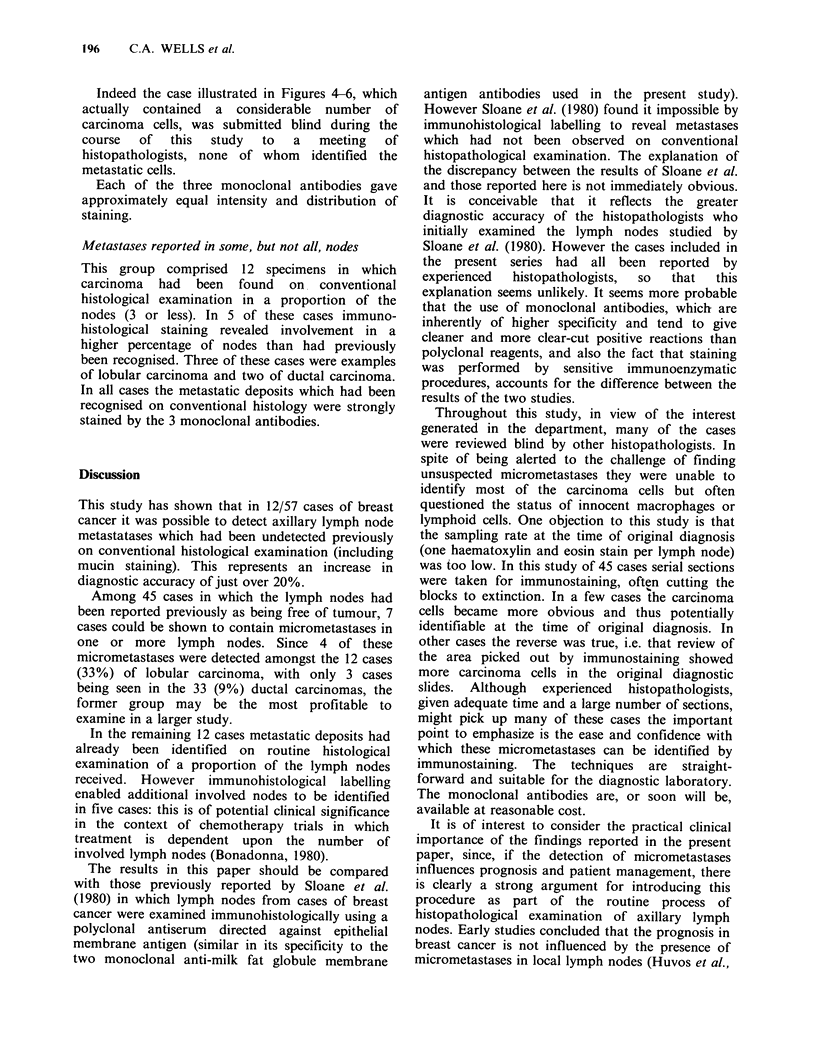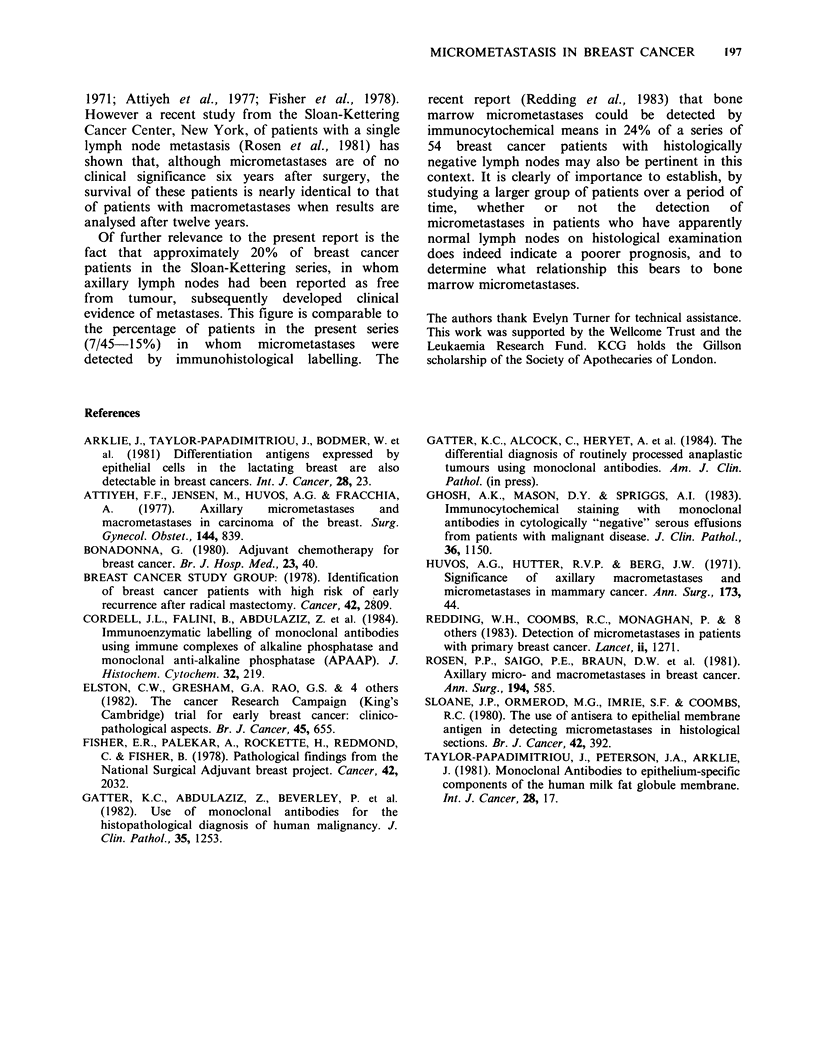# The immunocytochemical detection of axillary micrometastases in breast cancer.

**DOI:** 10.1038/bjc.1984.162

**Published:** 1984-08

**Authors:** C. A. Wells, A. Heryet, J. Brochier, K. C. Gatter, D. Y. Mason

## Abstract

**Images:**


*Br. J. Cancer* (1984) **50**, 193–197

### The immunocytochemical detection of axillary micrometastases in breast cancer

C.A. Wells, A. Heryet, J. Brochier[1], K.C. Gatter & D.Y. Mason

*Nuffield Department of Pathology, University of Oxford, John Radcliffe Hospital, Oxford, UK.*
[1]*Inserm 480, Hôpital Ed. – Herriot-PauP, 69374 Lyon Cedex 8, France.*

**Summary** The histological detection of tumour metastases in axillary lymph nodes from cases of breast carcinoma is of major prognostic significance, but may be difficult when metastases are of microscopic size. We have therefore investigated whether immunohistological techniques can increase the accuracy of metastasis detection in axillary lymph nodes. Forty-five cases of breast carcinoma were studied, in all of whom the axillary lymph nodes had been reported as free of metastases. Paraffin sections from these cases were stained by immunoenzymatic techniques, using monoclonal antibodies directed against human milk fat globule membrane antigen ("anti-EMA") and against epithelial intermediate filaments ("anti-keratin"). In 4/12 cases of lobular carcinoma and in 3/33 cases of ductal carcinoma, previously unsuspected micrometastases were revealed by immunohistological staining, representing an overall increase in detection rate of 15% (and of 33% for the lobular carcinoma cases). In addition to this group of 45 histologically "negative" biopsies, 12 samples were studied in which only a proportion of the nodes had been reported as containing tumour. In 5 of these cases immunostaining revealed previously undetected metastases. These findings suggest that immunohistological analysis may have a routine role to play in the staging of breast carcinoma. It is noted that the 15% increase in diagnostic accuracy achieved in the present study is comparable to the proportion of breast carcinoma patients in whom disseminated disease develops despite their axillary lymph nodes being reported as tumour-free at the time of surgery.

It is now widely accepted that involvement of axillary lymph nodes by breast cancer has an important bearing on prognosis (Breast Cancer Study Group, 1978; Elston *et al.*, 1982) and a decision on whether or not to undertake adjuvant chemotherapy frequently hinges on the number of involved nodes (Bonadonna, 1980). However, use of this criterion presupposes that micrometastases can reliably be detected by conventional histological procedures, e.g.: by examination of haematoxylin/eosin or mucin stains. A negative report therefore indicates that the histopathologist has been unable to identify any malignant cells, rather than that they are absent.

One potential means of improving observer accuracy in the detection of axillary micrometastases is to use immunocytochemical techniques. It has recently been reported by Redding *et al.* (1983) that small numbers of malignant cells may be detected by this approach in bone marrow samples from cases of human carcinoma when skeletal involvement is not evident from any other criteria. We have also recently reported that otherwise undetectable neoplastic cells can be demonstrated by immunocytochemical labelling in approximately 30% of cytologically "negative" serous effusions from cancer patients (Ghosh *et al.*, 1983).

We therefore undertook in the present study to assess whether immunocytological examination of axillary lymph nodes would offer any advantage over conventional histopathology in the detection of micrometastases.

Correspondence: K.C. Gatter.
Received 13 February 1984; accepted 13 April 1984.

### *Tissues*

All sections tested were prepared from routine formalin fixed paraffin embedded samples from the files of the Pathology Department of the John Radcliffe Hospital, Oxford. The specimens consisted of axillary lymph nodes removed at the time of surgery for breast carcinoma. The samples studied represented a random selection of mastectomy specimens received in the Department since 1980 in which axillary lymph nodes had been reported histologically as uninvolved by tumour, or in which only a proportion of the nodes were involved.

In Oxford there is no uniform surgical policy for sampling axillary lymph nodes. In this study, the average number of lymph nodes examined per case was 4, with the total number ranging from 1 to 11. Routine histological examination usually consists only of examination of one haematoxylin and eosin stained section per lymph node. Special stains such as Alcian blue-PAS had been performed originally only on a minority of cases in this study.

### *Monoclonal antibodies*

Three monoclonal antibodies were used in this investigation. Two were directed against human milk fat globule membrane antigens. One of these

© The Macmillan Press Ltd., 1984

(E29) was raised in our own laboratory (Gatter *et al.*, 1984), whilst the other (HMFG2) was produced in the ICRF Laboratories, London (Taylor-Papadimitriou *et al.*, 1981) and is obtainable from Seward Laboratory. Both antibodies were used as tissue culture supernatant diluted 1:10. The third antibody (KL1), kindly provided by Dr. J. Brochier, was raised against cytokeratins extracted from human epidermis. This antibody consisted of a purified immunoglobulin fraction and was diluted 1:100. The two anti-milk fat globule membrane antibodies have been screened in this laboratory in the past on normal and malignant tissues both in cryostat and paraffin embedded material (Arklie *et al.*, 1981; Gatter *et al.*, 1982, 1984). Antibody KL1 has been tested in its laboratory of origin on human tissues and found to stain most human epithelia but not non-epithelial cells or tissues.

*Immunoenzymatic reagents*

Peroxidase-conjugated rabbit antibodies to mouse Ig and swine antibodies to rabbit Ig were obtained from Dakopatts a/s. Alkaline phosphatase-conjugated anti-mouse Ig was kindly provided by Dr. K.-J. Pluzek. Diaminobenzidine tetrahydrochloride was obtained from Sigma Chemical Company. Tris buffered saline (TBS) was prepared by adding a tenth volume of 0.5 M Tris HCl buffer (pH 7.6) to 0.15 M saline.

*Immunoperoxidase staining*

Sections were prepared for staining by dewaxing and washing in TBS. Dewaxed sections were then incubated with monoclonal antibody for 30 min in a covered chamber. Sections were washed briefly in TBS and incubated for a further 30 min with peroxidase-conjugated rabbit anti-mouse Ig, diluted 1:50 in TBS to (which normal human serum has been added, at a final concentration of 1:2, in order to block cross-reactivity against human Ig). After washing in TBS, sections were incubated for a further 30 min with peroxidase-conjugated swine anti-rabbit Ig diluted 1:100 in TBS (containing normal human serum at a final concentration of 1:2).

Sections were then washed in TBS and the peroxidase reaction developed by incubating sections with TBS containing diaminobenzidine ($0.6 \mathrm{mg\,ml^{-1}}$) and $H_2O_2$ (0.01%) for 5–10 min at room temperature. Sections were washed in tap water, counterstained with haematoxylin and mounted for microscopical examination.

*Immuno-alkaline phosphatase labelling*

Some of the sections investigated in this study were analysed by a two-stage indirect immuno-alkaline phosphatase procedure. In this technique the initial stages up to and including incubation with the monoclonal antibody were performed as described above. Sections were then washed and incubated with alkaline phosphatase-conjugated rabbit anti-mouse Ig, diluted 1:20 in TBS containing 5% normal human serum. After a further wash the alkaline phosphatase reaction was developed using a substrate containing hexazotised new fuchsin and naphthol AS-MX, as described previously (Cordell *et al.*, 1984).

### Results

The results of this study are considered under two heads: firstly those cases in which no evidence of involvement by malignant cells was detected on routine histological examination in any of the nodes examined; and, secondly, those cases in which a proportion but not all of the nodes examined had been reported as containing metastatic carcinoma.

*Histologically negative nodes*

Axillary nodes from 45 cases of breast cancer (comprising 12 cases of lobular carcinoma and 33 of ductal carcinoma) which had previously been reported as ininvolved by tumour was examined immunocytochemically.

In 4 cases of lobular (33%) and 3 cases of ductal (9%) carcinoma micrometastases were revealed by histological staining.

Figures 1–6 illustrate the typical appearance of the immunocytochemical labelling reactions. It was noted that in most cases the tumour cells were present as single cells or small cell clusters in the sub-capsular or medullary sinuses (Figures 1–3) although in a few nodes widely disseminated (and previously unsuspected) neoplastic involvement throughout the substance of the node was revealed by immunohistological staining (Figures 5 and 6). No differences were seen in the distribution of occult tumour cells between lobular and ductal carcinomas.

H and E and Alcian blue-PAS stained adjacent sections from all cases in which immunohistological staining revealed the presence of previously undetected metastases were re-examined. It was possible to identify the malignant cells in most sections stained in this way, since the immunohistological labelling indicated precisely where they should be sought. However it should be emphasised that in most cases the number of carcinoma cells was low, and it was consequently only with the benefit of hindsight that neoplastic cells could be identified by H and E and Alcian blue-PAS staining.

**Figure 1** Immuno-peroxidase staining of a paraffin embedded axillary lymph node with monoclonal antibody E29 (anti-human milk fat globule membrane antigen). The biopsy had been reported on conventional histological examination as being free of tumour. However a strongly stained micrometastatic deposit is seen in the sub-capsular sinus (arrow).

**Figure 2** Haematoxylin and eosin stained section adjacent to that illustrated in **Figure 1**. The deposit of neoplastic cells in the sub-capsular sinus could be identified (arrow) by careful examination of the area in which the immunoperoxidase labelled cells had been seen.

**Figure 3** Immunoperoxidase detection of single neoplastic cells (arrows) in a sinus from an axillary lymph node which had been reported histologically as free of tumour. (Antibody E29).

**Figure 4** Low power photomicrpgraph of a haematoxylin and eosin stained axillary lymph node section in which metastases could not be detected, even on subsequent review, by a number of histopathologists.

**Figure 5** An adjacent section to **Figure 4** stained by the immuno-alkaline phosphatase method with the anti-keratin antibody KL1. It can clearly be seen at low power that there are a large number of infiltrating carcinoma cells present not only in the thickened capsule but also within the substance of the lymph node itself.

**Figure 6** A high power field from the case illustrated in **Figure 4** stained for milk fat globule membrane antigen (antibody E29) using an immunoperoxidase technique. The neoplastic cells are characterised by the presence of intracytoplasmic lumina (arrows). These structures could be identified in Alcian blue-PAS stained sections (although only when the sites of neoplastic cell infiltration had been identified by immunohistological labelling).

Indeed the case illustrated in Figures 4–6, which actually contained a considerable number of carcinoma cells, was submitted blind during the course of this study to a meeting of histopathologists, none of whom identified the metastatic cells.

Each of the three monoclonal antibodies gave approximately equal intensity and distribution of staining.

*Metastases reported in some, but not all, nodes*

This group comprised 12 specimens in which carcinoma had been found on conventional histological examination in a proportion of the nodes (3 or less). In 5 of these cases immunohistological staining revealed involvement in a higher percentage of nodes than had previously been recognised. Three of these cases were examples of lobular carcinoma and two of ductal carcinoma. In all cases the metastatic deposits which had been recognised on conventional histology were strongly stained by the 3 monoclonal antibodies.

### Discussion

This study has shown that in 12/57 cases of breast cancer it was possible to detect axillary lymph node metastatases which had been undetected previously on conventional histological examination (including mucin staining). This represents an increase in diagnostic accuracy of just over 20%.

Among 45 cases in which the lymph nodes had been reported previously as being free of tumour, 7 cases could be shown to contain micrometastases in one or more lymph nodes. Since 4 of these micrometastases were detected amongst the 12 cases (33%) of lobular carcinoma, with only 3 cases being seen in the 33 (9%) ductal carcinomas, the former group may be the most profitable to examine in a larger study.

In the remaining 12 cases metastatic deposits had already been identified on routine histological examination of a proportion of the lymph nodes received. However immunohistological labelling enabled additional involved nodes to be identified in five cases: this is of potential clinical significance in the context of chemotherapy trials in which treatment is dependent upon the number of involved lymph nodes (Bonadonna, 1980).

The results in this paper should be compared with those previously reported by Sloane *et al.* (1980) in which lymph nodes from cases of breast cancer were examined immunohistologically using a polyclonal antiserum directed against epithelial membrane antigen (similar in its specificity to the two monoclonal anti-milk fat globule membrane antigen antibodies used in the present study). However Sloane *et al.* (1980) found it impossible by immunohistological labelling to reveal metastases which had not been observed on conventional histopathological examination. The explanation of the discrepancy between the results of Sloane *et al.* and those reported here is not immediately obvious. It is conceivable that it reflects the greater diagnostic accuracy of the histopathologists who initially examined the lymph nodes studied by Sloane *et al.* (1980). However the cases included in the present series had all been reported by experienced histopathologists, so that this explanation seems unlikely. It seems more probable that the use of monoclonal antibodies, which are inherently of higher specificity and tend to give cleaner and more clear-cut positive reactions than polyclonal reagents, and also the fact that staining was performed by sensitive immunoenzymatic procedures, accounts for the difference between the results of the two studies.

Throughout this study, in view of the interest generated in the department, many of the cases were reviewed blind by other histopathologists. In spite of being alerted to the challenge of finding unsuspected micrometastases they were unable to identify most of the carcinoma cells but often questioned the status of innocent macrophages or lymphoid cells. One objection to this study is that the sampling rate at the time of original diagnosis (one haematoxylin and eosin stain per lymph node) was too low. In this study of 45 cases serial sections were taken for immunostaining, often cutting the blocks to extinction. In a few cases the carcinoma cells became more obvious and thus potentially identifiable at the time of original diagnosis. In other cases the reverse was true, i.e. that review of the area picked out by immunostaining showed more carcinoma cells in the original diagnostic slides. Although experienced histopathologists, given adequate time and a large number of sections, might pick up many of these cases the important point to emphasize is the ease and confidence with which these micrometastases can be identified by immunostaining. The techniques are straightforward and suitable for the diagnostic laboratory. The monoclonal antibodies are, or soon will be, available at reasonable cost.

It is of interest to consider the practical clinical importance of the findings reported in the present paper, since, if the detection of micrometastases influences prognosis and patient management, there is clearly a strong argument for introducing this procedure as part of the routine process of histopathological examination of axillary lymph nodes. Early studies concluded that the prognosis in breast cancer is not influenced by the presence of micrometastases in local lymph nodes (Huvos *et al.*,

1971; Attiyeh *et al.*, 1977; Fisher *et al.*, 1978). However a recent study from the Sloan-Kettering Cancer Center, New York, of patients with a single lymph node metastasis (Rosen *et al.*, 1981) has shown that, although micrometastases are of no clinical significance six years after surgery, the survival of these patients is nearly identical to that of patients with macrometastases when results are analysed after twelve years.

Of further relevance to the present report is the fact that approximately 20% of breast cancer patients in the Sloan-Kettering series, in whom axillary lymph nodes had been reported as free from tumour, subsequently developed clinical evidence of metastases. This figure is comparable to the percentage of patients in the present series (7/45—15%) in whom micrometastases were detected by immunohistological labelling. The recent report (Redding *et al.*, 1983) that bone marrow micrometastases could be detected by immunocytochemical means in 24% of a series of 54 breast cancer patients with histologically negative lymph nodes may also be pertinent in this context. It is clearly of importance to establish, by studying a larger group of patients over a period of time, whether or not the detection of micrometastases in patients who have apparently normal lymph nodes on histological examination does indeed indicate a poorer prognosis, and to determine what relationship this bears to bone marrow micrometastases.

The authors thank Evelyn Turner for technical assistance. This work was supported by the Wellcome Trust and the Leukaemia Research Fund. KCG holds the Gillson scholarship of the Society of Apothecaries of London.

### References

ARKLIE, J., TAYLOR-PAPADIMITRIOU, J., BODMER, W. et al. (1981) Differentiation antigens expressed by epithelial cells in the lactating breast are also detectable in breast cancers. *Int. J. Cancer*, **28**, 23.

ATTIYEH, F.F., JENSEN, M., HUVOS, A.G. & FRACCHIA, A. (1977). Axillary micrometastases and macrometastases in carcinoma of the breast. *Surg. Gynecol. Obstet.*, **144**, 839.

BONADONNA, G. (1980). Adjuvant chemotherapy for breast cancer. *Br. J. Hosp. Med.*, **23**, 40.

BREAST CANCER STUDY GROUP: (1978). Identification of breast cancer patients with high risk of early recurrence after radical mastectomy. *Cancer*, **42**, 2809.

CORDELL, J.L., FALINI, B., ABDULAZIZ, Z. et al. (1984). Immunoenzymatic labelling of monoclonal antibodies using immune complexes of alkaline phosphatase and monoclonal anti-alkaline phosphatase (APAAP). *J. Histochem. Cytochem.* **32**, 219.

ELSTON, C.W., GRESHAM, G.A. RAO, G.S. & 4 others (1982). The cancer Research Campaign (King's Cambridge) trial for early breast cancer: clinico-pathological aspects. *Br. J. Cancer*, **45**, 655.

FISHER, E.R., PALEKAR, A., ROCKETTE, H., REDMOND, C. & FISHER, B. (1978). Pathological findings from the National Surgical Adjuvant breast project. *Cancer*, **42**, 2032.

GATTER, K.C., ABDULAZIZ, Z., BEVERLEY, P. et al. (1982). Use of monoclonal antibodies for the histopathological diagnosis of human malignancy. *J. Clin. Pathol.*, **35**, 1253.

GATTER, K.C., ALCOCK, C., HERYET, A. et al. (1984). The differential diagnosis of routinely processed anaplastic tumours using monoclonal antibodies. *Am. J. Clin. Pathol.* (in press).

GHOSH, A.K., MASON, D.Y. & SPRIGGS, A.I. (1983). Immunocytochemical staining with monoclonal antibodies in cytologically "negative" serous effusions from patients with malignant disease. *J. Clin. Pathol.*, **36**, 1150.

HUVOS, A.G., HUTTER, R.V.P. & BERG, J.W. (1971). Significance of axillary macrometastases and micrometastases in mammary cancer. *Ann. Surg.*, **173**, 44.

REDDING, W.H., COOMBS, R.C., MONAGHAN, P. & 8 others (1983). Detection of micrometastases in patients with primary breast cancer. *Lancet*, **ii**, 1271.

ROSEN, P.P., SAIGO, P.E., BRAUN, D.W. et al. (1981). Axillary micro- and macrometastases in breast cancer. *Ann. Surg.*, **194**, 585.

SLOANE, J.P., ORMEROD, M.G., IMRIE, S.F. & COOMBS, R.C. (1980). The use of antisera to epithelial membrane antigen in detecting micrometastases in histological sections. *Br. J. Cancer*, **42**, 392.

TAYLOR-PAPADIMITRIOU, J., PETERSON, J.A., ARKLIE, J. (1981). Monoclonal Antibodies to epithelium-specific components of the human milk fat globule membrane. *Int. J. Cancer*, **28**, 17.